# A Miniature Soft Sensor with Origami-Inspired Self-Folding Parallel Mechanism

**DOI:** 10.3390/mi13081188

**Published:** 2022-07-28

**Authors:** Yongqi Shi, Gang Wang, Wenguang Sun, Yunfeng Ya, Shuhan Liu, Jiongjie Fang, Feiyang Yuan, Youning Duo, Li Wen

**Affiliations:** 1 School of Mechanical Engineering and Automation, Beihang University, Beijing 100191, China; 15810960617@163.com (Y.S.); charleswang0226@yahoo.com (G.W.); buaaswg@126.com (W.S.); pinkcc2003@163.com (Y.Y.); buaalsh@163.com (S.L.); 19374471@buaa.edu.cn (J.F.); feiyangyuan@buaa.edu.cn (F.Y.); dyn@buaa.edu.cn (Y.D.); 2 Beijing Advanced Innovation Center for Biomedical Engineering, Beihang University, Beijing 100191, China

**Keywords:** millimeter scale, soft sensor, origami, self-folding

## Abstract

Miniature soft sensors are crucial for the perception of soft robots. Although centimeter-scale sensors have been well developed, very few works addressed millimeter-scale, three-dimensional-shaped soft sensors capable of measuring multi-axis forces. In this work, we developed a millimeter-scale (overall size of 6 mm × 11 mm × 11 mm) soft sensor based on liquid metal printing technology and self-folding origami parallel mechanism. The origami design of the sensor enables the soft sensor to be manufactured within the plane and then fold into a three-dimensional shape. Furthermore, the parallel mechanism allows the sensor to rotate along two orthogonal axes. We showed that the soft sensor can be self-folded (took 17 s) using a shape-memory polymer and magnets. The results also showed that the sensor prototype can reach a deformation of up to 20 mm at the tip. The sensor can realize a measurement of external loads in six directions. We also showed that the soft sensor enables underwater sensing with a minimum sensitivity of 20 mm/s water flow. This work may provide a new manufacturing method and insight into future millimeter-scale soft sensors for bio-inspired robots.

## 1. Introduction

Miniature soft robots have a promising future because of their compact size, high mobility, and precision manipulation [1,2]. Those properties enable a wide range of applications, such as handling micro objects [3,4], minimally invasive surgery [5], and environmental exploration [6]. Sensing is crucial for soft robots to perceive their movements and surrounding environment [7,8]. Nevertheless, sensors used for miniature soft robots often require unconventional approaches [9]. For example, Liu et al. integrated the liquid metal sensing layer on the soft robot to sense the motion state of the robot [10]. Firouzeh et al. used carbon ink sensors to measure the rotational angle of shape memory alloy [11]. Moreover, commercial alloy strain sensors [12], smart materials [13,14], and other sensing schemes [15] were also adopted. However, most researchers focused on proprioception, and the capability of miniature soft robots to perceive the surrounding environment is relatively weak. As a result, a compact, adaptable, resilient miniature soft sensor with multi-axis force and strain sensing capabilities remains to be investigated.

The challenges of designing an ideal sensor are primarily about the trade-off between sensor scale, resilience, sensitivity, and manufacturing. Gafford et al. built a millimeter-scale uniaxial force sensor and a 3-axis force sensor based on the principle of light intensity modulation and micro strain gauge, respectively, [16,17]. They are within 20 mm in size, simple to manufacture, and the measurement accuracy reaches the millinewton level. The former can even realize self-assembling, but both are made of rigid materials and have limited ability regarding overload. Most rigid miniature sensors based on other transducer mechanisms have the same limitations [18].

On the other hand, one typical soft sensing approach is using highly conductive liquid (e.g., EGaIn [19,20], conductive ion solutions [21]) as resistance. That type of soft sensor is highly deformable and sensitive; however, sensing multi-directional force remains a challenge. Park et al. developed a soft artificial skin composed of three layers of liquid metal micro-channels [22,23]. The sensor size is 25 mm × 25 mm with a thickness of 3.5 mm. This prototype can sense force in three directions. Furthermore, researchers also used independent liquid metal channels to realize multi-directional force sensing [24,25]. They put a rigid pole onto the soft material. The sensor signal will change when the pole transmits the pressure onto the liquid metal channel. However, interference between different liquid metal channels exists, thus compromising the sensitivity.

The silicon-based Micro-Electro-Mechanical Systems (MEMS) technique has provided a solution for sub-millimeter scale manufacturing. However, few studies addressed fabricating miniature scale sensors (<10 mm) [26]. These soft sensors are usually challenging to build, requiring multiple steps in the complex manufacturing process. One development of the miniature structure fabrication technique was printed circuit MEMS (PC-MEMS) [27]. Inspired by printed circuit board (PCB) manufacturing and origami concept, PC-MEMS was developed. A complex three-dimensional structure can be fabricated through four basic operations: additive lamination, subtractive micromachining, folding, and locking. Although this solved the problem of manufacturing on a millimeter to centimeter scale, the folding process still requires hand-made, which is time-consuming and low efficient. Raised up by the same research group, one solution is the Pop-up Book MEMS technique which was inspired by the design of pop-up books [26]. It can use a single degree-of-freedom mechanism to perform the assembly process of a complex structure. One needs to convert the originally multi-degrees-of-freedom assembly process into a single-degree-of-freedom assembly process by adding hinges. All designs are hand-crafted, which is tedious and error-prone. The self-folding technique is also an effective way to simplify the fabrication process [28]. Automatic assembly can achieve structural assembly from planar to three-dimensional, via external stimulation measures, such as light [29], heat [30], electric field [28,31], or PH value of a solution [32]. Automatic assembly is essential for millimeter-scale sensors because it could simplify the complicated hand-made process [33]. Some progress has been made in millimeter-scale manufacturing, nevertheless, they lack sensing ability [34,35].

The origami concept not only inspires the fabrication technique of miniature structures, but also gives researchers the flexibility to design three-dimensional structures with rich configurations and diverse functions from flat patterns [36]. Origami and kirigami are two related but different concepts. One view is that kirigami includes both the processes of “cutting” and “folding”, while origami only contains the latter [37]. In contrast, other researchers believe that cutting can also be used in origami to change the initial shape of the paper, also known as pre-patterning procedures [38]. According to previous research, in many studies, there is no clear boundary between origami and kirigami [37]. Our work derived from the former study which refers to origami-inspired [28,30,31], so we chose to use the term “origami” for consistency.

**Figure 1:**
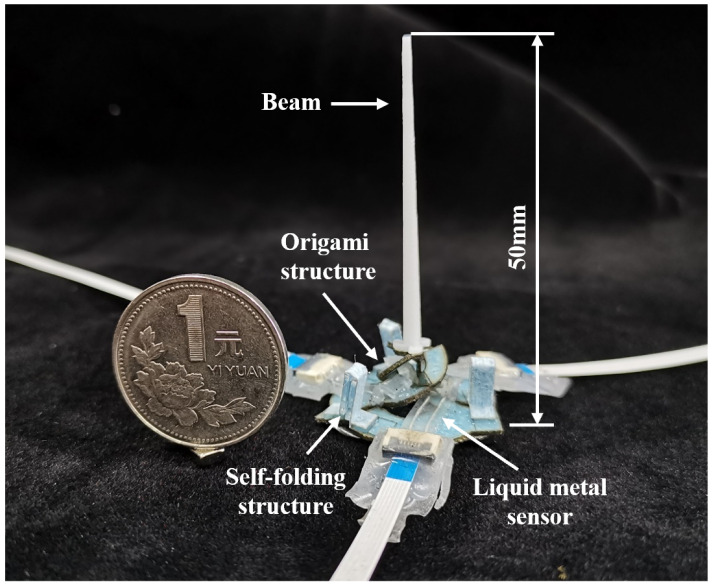
A miniature soft sensor with origami-inspired self-folding parallel mechanism. The coin’s diameter is 25 mm.

In this paper, we aim to implement a miniature soft sensor with an origami-inspired self-folding parallel mechanism (Figure 1). First, we introduced the millimeter-scale sensor and the fabrication process using the self-folding technique, helping the sensor self-assemble under heating stimuli. Then, a kinematic model was established, which described the relationship between the sensor signal and the external load. Finally, experiments were conducted to test the capabilities of the sensor. We further demonstrated the sensing ability underwater, proving its wide range of possible applications.

## 2. Materials and Methods

### 2.1. Miniature Soft Sensor Overview

**Figure 2:**
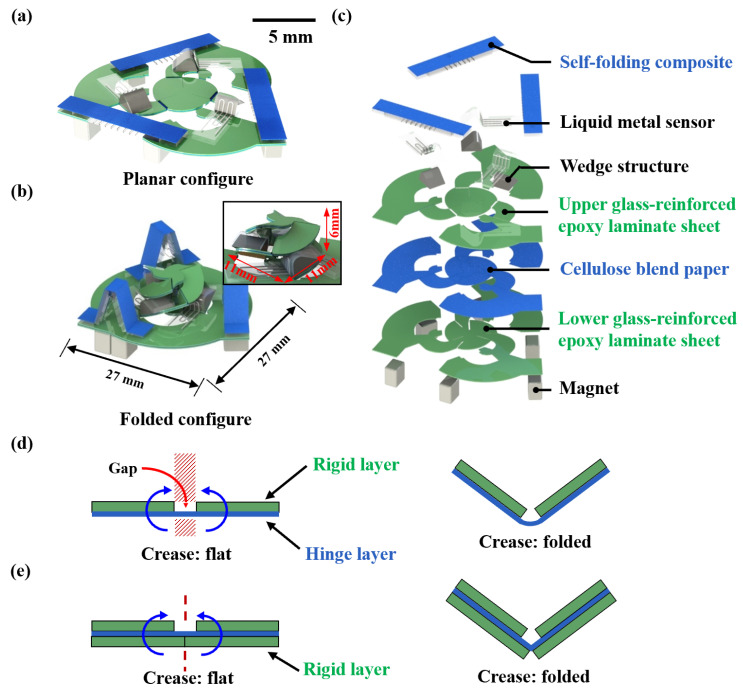
Design of the miniature soft sensor. (**a**) Flat state configuration. (**b**) Folded configuration. The inset is an enlarged view of the origami structure with the liquid metal sensor. (**c**) Explosion diagram of the miniature soft sensor. The self-folding composite and the liquid metal sensor’s thicknesses are 900 μm and 600 μm. The thickness of the upper and lower glass-reinforced epoxy laminate sheet and the cellulose blend paper is 200 μm. (**d**) An origami structure with a single rigid layer. The red shaded part represents the possible position of the rotating shaft. (**e**) An origami structure with double rigid layers.

Inspired by origami structures, a miniature soft sensor was designed. The sensor dimension is 6 mm × 11 mm × 11 mm. The diameter of the sensor base is 27 mm. The miniature soft sensor has two configurations, planar configure and folded configure, as shown in Figure 2a,b. In the fabrication process, we used the planar fabrication technique to make each layer of the planar configured sensor. Then, by using layer-up and self-folding techniques, the sensor configuration could change into folded configuration and remain a final state for sensing. The miniature soft sensor is made of seven layers (Figure 2c). The sensor can be categorized into three parts: self-folding units made of self-folding composite and micro magnets, sensing units made of liquid metal sensors and wedge structures, and a miniature origami structure made of glass-reinforced epoxy laminate sheet and cellulose blend paper.

In the miniature origami structure, the layers of glass-reinforced epoxy laminate sheet are also called the rigid layer, and the layer of cellulose blend paper is also called the hinge layer, which is flexible but cannot stretch. Both the rigid layers and the hinge layer have a thickness of 200 μm. The miniature origami structure consists of 12 creases, and each crease is equal to a single-degree-of-freedom revolute joint. The area between two hinges can be seen as an arm. For a single crease, it is significant to ensure the position and orientation of the rotation axis stay stationary as the joint moves. The width of the revolute joint that suffices the 180 deg rotation between two linkages causes undesired out-of-plane rotations. In order to prevent this problem, we used double rigid layers instead of a typical single layer. Figure 2d,e shows the difference between the two solutions. Double layers can restrain the axis to a fixed position compared to the single rigid layer. Meanwhile, we can adjust the fold angle by varying the gap width of the rigid layers. The rigid layer acts as a mechanical stop, and a greater gap allows for a larger fold angle.

Three soft sensing units were distributed at the sensor base’s crease to measure the crease’s rotation angle. The sensing unit consisted of the liquid metal sensing part (200 μm in thickness) and the wedge structure (2.25 mm in height). The liquid metal sensing part was fabricated by molded silicone elastomer (Ecoflex 00-30, Smooth-on, Inc., Macungie, PA, USA). Inside the soft material, the micro-channel was filled with liquid metal. When the crease of the origami structure was folded, the liquid metal micro-channel was pressed, so the corresponding resistance increased. The wedge structure was designed to perform preload on the liquid metal micro-channel to increase sensor sensitivity. Compared to traditional strain sensing foil, a soft sensor based on liquid metal allows the sensing unit to deform without movement restriction.

The self-folding unit is based on a heat-sensitive sheet and a micro NdFeB magnet (size: 4 mm × 4 mm × 3 mm). The heat-sensitive sheet is a shape-memory polymer mechanically programmed to contract bidirectionally when heated to approximately 140 °C via electrical current. The miniature soft sensor can self-assemble from planar to the three-dimensional mode by actuating three self-folding units. Overall, the miniature soft sensor uses a high-precision planar manufacturing process, which allows mass production and self-assembly by actuating self-folding units. When the sensor perceives loads from different directions, the origami structure will cause the corresponding crease to fold or stretch. By measuring the sensing unit signal, the rotation angle could be characterized. Furthermore, the sensor posture could be acquired with the kinematic model, then the relationship between sensor beam tip displacement and sensor signal can be found.

### 2.2. Fabrication

**Figure 3:**
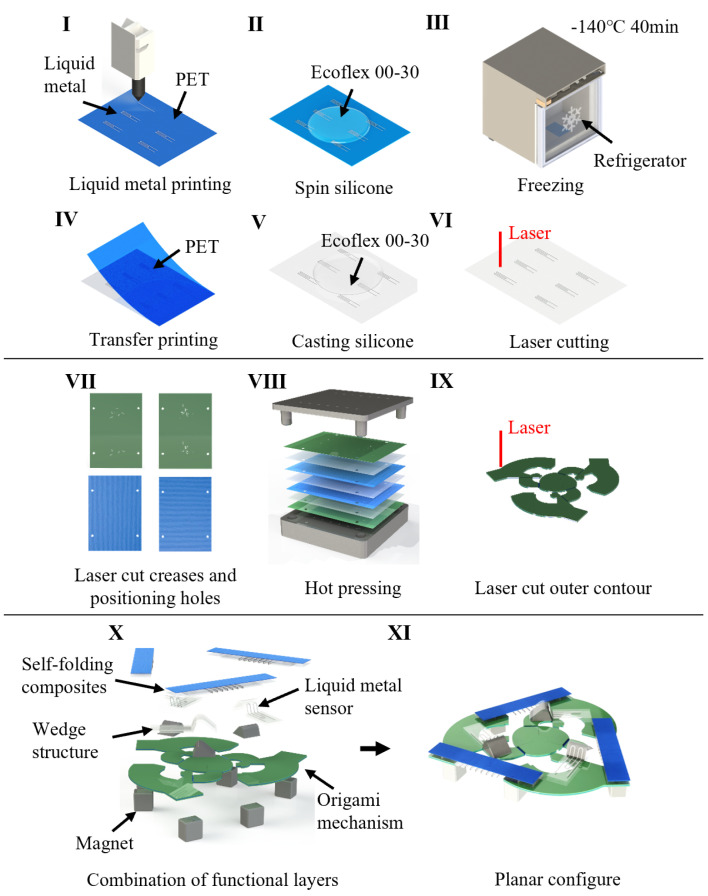
Fabrication process of the miniature soft sensor. I–VI shows the fabrication of liquid metal sensors. VII–IX shows the fabrication of the micro origami mechanism. X–XI shows the combination of each functional layer and forming of the overall structure.

Sensor fabrication can be divided into three parts, miniature origami structure, sensing units, and self-folding units. Each part was fabricated individually and assembled, as shown in Figure 3.

The origami structure was made by laser cutting technology, using a CO${}_{2}$ laser system (VLS 2.3, Universal Laser Systems). First, we cut a 200 μm thick glass-reinforced epoxy laminate sheet (FR-4) into a 100 mm × 70 mm rectangle, along with four dowel holes and each crease. As in the first step, we cut dowel holes on two pieces of 100 μm thick cellulose blend paper (ENLES, Suzhou, Jiangsu, China) and three pieces of 50 μm thick hot melt adhesive film (tesa HAF^®^ 8402, tesa Inc., Hamburg, Germany). The cellulose blend paper has fiber directions. The tensile strength is lower perpendicular to the fiber direction, while the tensile strength is about 30 times higher along the fiber direction. We kept the fiber directions of two pieces of cellulose blend paper orthogonal to obtain an excellent tensile strength in both directions. Then, a heat press was used to bond the rigid layer (fiberglass) and the hinge layer (blend paper). A set of positioning apparatuses were used to ensure the relative positioning of each layer was within the error of 200 μm. During hot press, we used precision dowels and a set of positioning apparatuses to ensure positioning of each layer, and set the temperature to 200 °C and timer for 180 s. After hot press, a second laser cutting was the final process for the origami structure.

Fabrication of the sensing units is based on liquid metal printing technology. After designing a liquid metal pattern in computer aided design software, we loaded the dxf file onto the liquid metal printer (Dream S1, Dream-Inc Inc., Beijing, China). The liquid metal printer has the capability to print liquid metal onto a plastic film. Next, silicon elastomer (Ecoflex 00-30, Smooth-on Inc., Macungie, PA, USA) was mixed at a weight ratio of 1:1, and degassed in a vacuum chamber for 5 min, then poured onto the plastic film with the liquid metal pattern. After spin coating at 100 rpm for 60 s, the plastic film was put into an industrial freezer with a temperature of −140 °C for 40 min. Then it was severed from the soft material. We manually soldered a flat wire socket with wire onto the liquid metal with a soldering kit and poured another layer of silicone elastomer to cover the surface of the liquid metal. After spin coating at 250 rpm for 60 s, laser cutting was used to shape the outline of the sensing units. Wedge structures were made of 4 mm thick acrylic plate, and were fabricated by laser. They have triangular cross sections with a vortex angle of 65 degrees and a height of 2.25 mm.

Fabrication of the self-folding composites is also based on laser cutting. First, the creases were cut on a 500 μm thick glass-reinforced epoxy laminate sheet. Next, we used a heat press to bond the fiberglass and the blend paper. Then, a laser was used to cut the outline. Finally, we used glue (Loctite 401, Hanke, Dusseldorf, North Rhine-Westphalia, Germany) to stick a heat-sensitive sheet (Poly-Vinyl Chloride; PVC, Shrink Bag, shrinking temperature: 120 °C–150 °C, thickness 300 μm) bonded with nichrome wire (diameter 100 μm) onto the former part.

The assembly process is called ‘pick-and-place’. We placed the sensing units, the self-folding composites and the N35H NdFeB magnets onto the corresponding position using silicon rubber adhesive (Smooth-on Sil-Poxy, Smooth-on Inc., Macungie, PA, USA) and glue (Loctite 401, Hanke, Dusseldorf, North Rhine-Westphalia, Germany). Then we applied electrical current to the nichrome wire, and the heat-sensitive sheet would shrink and initiate the self-folding process. After the self-folding process, the sensor was successfully fabricated.

### 2.3. Sensor Modeling and Kinematics

In this section, we describe the mechanical configuration of the sensor in detail and then establish a kinematic model of the sensor.

**Figure 4:**
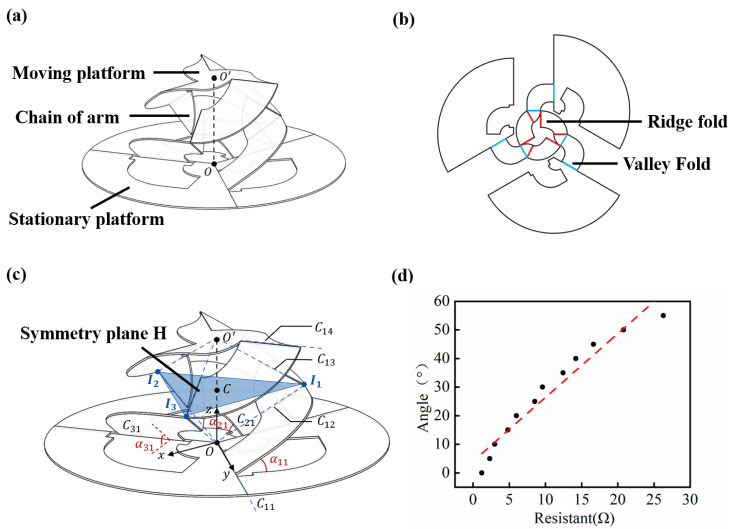
Sensor modeling and kinematics. (**a**) Schematic diagram of the three-dimensional mechanism of the miniature soft sensor. (**b**) Crease pattern of the miniature soft sensor, in which the blue line represents the folding method of folding forward to form a valley shape, and the red line represents the folding method of folding backward to form a mountain shape. (**c**) Schematic diagram of the mechanism with labels. (**d**) The relationship between the crease rotation angle and the resistance of the sensing unit.

Inspired by the parallel mechanism Omni-Wrist III [39], a new three-dimensional mechanism was designed and introduced. As shown in Figure 4a,b, from a mechanism standpoint, the sensor consists of a moving platform, a stationary platform, and three chains of arms distributed equally on the circumference. Each crease can be equal to a revolute joint, and the area between two creases can equal an arm. As with Omni-Wrist III, our miniature soft sensor can rotate along two orthogonal axes. The center of the moving platform O’ can move on a spherical surface.

As shown in Figure 4c, $C_{i1}∼C_{i4}\;\left(i=1,2,3\right)$ denotes four creases of chain *i*, from bottom to the top. *O* denotes the center of the stationary platform, intersected at crease extended line $C_{i1}$ and $C_{i2}$. $O^{{}^{\prime }}$ denotes the center of the stationary platform, intersected at crease extended line $C_{i3}$ and $C_{i4}$. $I_{i}$ denotes the intersection point of creases extended line $C_{i2}$ and $C_{i3}$. In the research of Yu et al. [40], this type of mechanism always has a symmetry plane H which is vertical to ${\mathrm{OO}}^{{}^{\prime }}$. During the movement, the upper and lower parts of the mechanism are always symmetrical about plane H. Plane H can be determined from the center point *C* of ${\mathrm{OO}}^{{}^{\prime }}$, and $I_{i}$.

The coordinate system Oxyz was established on the base platform. The *z*-axis direction is vertical and pointing upward. The *y*-axis direction is determined by crease $C_{i1}$, and the *x*-axis direction is determined from the right-hand screw rule. The kinematic equation is described as follows:

(1) $$\left(x,y,z\right)=f\left(\alpha_{11},\alpha_{21},\alpha_{31}\right)$$

where $\left(x,y,z\right)$ denotes the coordinate of moving platform center point $O^{{}^{\prime }}$, and $\left(\alpha_{11},\alpha_{21},\alpha_{31}\right)$ denotes the rotation angle of rotating pairs $C_{11}$, $C_{21}$, $C_{31}$, respectively.

Due to the symmetry of the mechanism, the connecting line between the center points of the moving platform and the base platform, namely ${\mathrm{OO}}^{{}^{\prime }}$, is always perpendicular to ${\mathrm{CI}}_{i}$, where *C* is the intersection of ${\mathrm{OO}}^{{}^{\prime }}$ and the symmetry plane H. The following geometric constraint equations can be obtained accordingly:

(2) $$\left\{\begin{array}{c} {\mathrm{OO}}^{{}^{\prime }}\cdot {\mathrm{CI}}_{1}=0\\ {\mathrm{OO}}^{{}^{\prime }}\cdot {\mathrm{CI}}_{2}=0\\ {\mathrm{OO}}^{{}^{\prime }}\cdot {\mathrm{CI}}_{3}=0 \end{array}\right.$$

Because of ${\mathrm{OO}}^{{}^{\prime }}={\left(x,y,z\right)}^{T}$, ${\mathrm{CI}}_{i}\;\left(i=1,2,3\right)$ can be solved as follows:

(3) $$\left\{\begin{array}{c} {\mathrm{CI}}_{i}={\mathrm{OI}}_{i}-\mathrm{OC}\\ {\mathrm{OI}}_{i}=R_{z}\left(\beta_{i}\right)R_{z}\left(90^{\circ }\right)R_{x}\left(\alpha_{i1}\right){\left(0,L,0\right)}^{T} \end{array}\right.$$

where $\beta_{i}\;\left(i=1,2,3\right)$ are parameters describing the spatial position of each chains, specifically, $\beta_{1}=0^{\circ },\beta_{2}=120^{\circ },\beta_{3}=240^{\circ }$. $R_{z}\left(90^{\circ }\right)$ is the rotation matrix to rotate 90 degrees around the *z*-axis; $R_{x}\left(\alpha_{i1}\right)$ is the rotation matrix to rotate an angle α around the *x*-axis; *L* is the length of ${\mathrm{OI}}_{i}\;\left(i=1,2,3\right)$.

This leads to:

(4) $${\mathrm{CI}}_{i}=\left(\begin{array}{c} -L\cos\alpha_{i1}\cos\beta_{i}-\frac{x}{2}\\ -L\cos\alpha_{i1}\sin\beta_{i}-\frac{y}{2}\\ L\sin\alpha_{i1}-\frac{z}{2} \end{array}\right)$$

Substitute Equation (4) into Equation (2) and simplify it using $x^{2}+y^{2}+z^{2}=R^{2}$:

(5) $$\left\{\begin{array}{cc} \; & -L\cos\alpha_{11}\cos\beta_{1}x-L\cos\alpha_{11}\sin\beta_{1}y+L\sin\alpha_{11}z=\frac{1}{2}R^{2}\\ \; & -L\cos\alpha_{21}\cos\beta_{2}x-L\cos\alpha_{21}\sin\beta_{2}y+L\sin\alpha_{21}z=\frac{1}{2}R^{2}\\ \; & -L\cos\alpha_{31}\cos\beta_{3}x-L\cos\alpha_{31}\sin\beta_{3}y+L\sin\alpha_{31}z=\frac{1}{2}R^{2} \end{array}\right.$$

where *R* is the length of ${\mathrm{OO}}^{{}^{\prime }}$.

Let $A=L\cdot \left(\begin{array}{ccc} -\cos\alpha_{11}\cos\beta_{1} & -\cos\alpha_{11}\sin\beta_{1} & \sin\alpha_{11}\\ -\cos\alpha_{21}\cos\beta_{2} & -\cos\alpha_{21}\sin\beta_{2} & \sin\alpha_{21}\\ -\cos\alpha_{31}\cos\beta_{3} & -\cos\alpha_{31}\sin\beta_{3} & \sin\alpha_{31} \end{array}\right),B=\left(\begin{array}{c} x\\ y\\ z \end{array}\right),C=\frac{1}{2}R^{2}\left(\begin{array}{c} 1\\ 1\\ 1 \end{array}\right)$, the above formula can be expressed as:

(6) A·B=C

When $\left|A\neq 0\right|$, it can be solved:

(7) $$B=A^{-1}C$$

Finally, by substituting the values in Table 1 into Equation (7), the specific mathematical expression between the $O^{{}^{\prime }}$ point coordinates $\left(x,y,z\right)$ and the rotation angle $\left(\alpha_{11},\alpha_{21},\alpha_{31}\right)$ can be obtained.

After adding constraints, using the Monte Carlo method to analyze the working space of the mechanism according to the sensor kinematic model, it can be obtained that the normal vector of the moving platform moves in a cone with a cone angle of 120°.

The different posture of the sensor reflects the magnitude and direction of the external load. By establishing the kinematic model, we found the relationship between the sensing posture and the crease rotation angle but lacked the corresponding relationship between the crease rotation angle and the sensor signal. Therefore, we characterized the soft sensing unit, which was fixed at a crease, slowly turning one side of the crease, and used a magnetic encoder and a precision multimeter (Fluke 8845A, Fluke Inc., Everett, Washington, DC, USA) to record the change of rotation angle and the change of liquid metal resistance, respectively. As shown in Figure 4d, we measured 12 data points that were linearly fitted. The relationship between the crease rotation angle and the resistance of the sensing unit was as follows:

(8) $$\alpha =2.364\times r+3.015\;\;\;\;(R^{2}=0.95)$$

where α is the crease rotation angle, *r* is the sensor resistance. $R^{2}$ of the linear regression is 0.95.

When applied displacement to the sensor, the sensor signal is measured, and according to the amplified parameters, we can obtain the rotation angle according to Equation (8). Finally, we can solve the posture of the moving platform according to the kinematic model, so that the amplitude and the direction of the external load can be found.

### 2.4. Self-Folding of the Sensor

We designed a millimeter-scale self-folding technique based on a heat-sensitive sheet and micro magnets, which enabled the remote self-assembling of the sensor. The heat sensitive sheet is a shape memory polymer (Poly-Vinyl Chloride; PVC, Shrink Bag). It retracts in opposite directions after heating, which can provide initial assembly displacement to trigger a further attracting movement of the permanent magnets to realize self-assembly.

**Figure 5:**
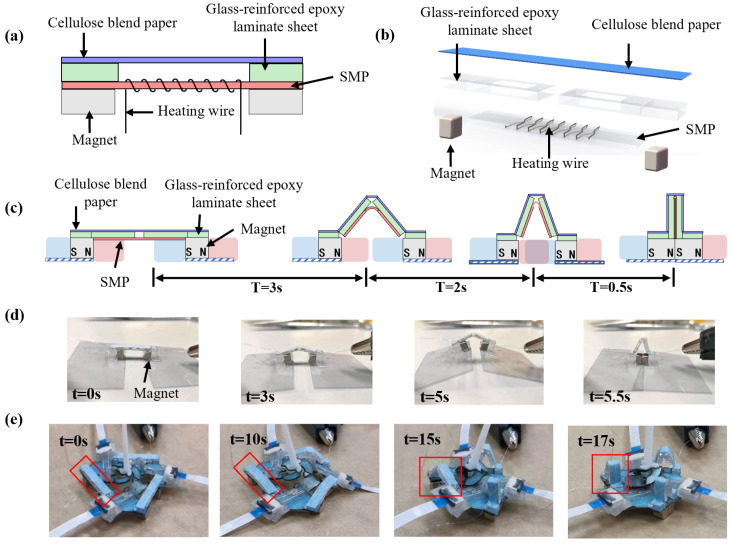
Design of millimeter-scale self-folding technique. (**a**) Structural composition of a single self-folding unit. (**b**) Structural exploded view of a single self-folding unit. (**c**) Schematic diagram of the self-folding process. T indicates the time required for the transition period. (**d**) Photos of the self-folding process. (**e**) Photos of the self-folding process of the miniature soft sensor. The red rectangle shows the movement process of one self-folding unit. See Video S1 for more details.

This technique used three identical self-folding units to assemble the three sectors in Figure 4b. Due to the flexibility and elasticity at the crease, the sensor can pop up from the plane state to the three-dimensional state. Figure 5a,b shows the composition of a single self-folding unit. From top to bottom, it consists of a single degree of freedom origami mechanism composed of a hinge layer and a rigid layer, a heat-sensitive sheet (Poly-Vinyl Chloride; shrinking temperature: 120 °C–150 °C thickness 300 μm), a nickel-chromium alloy wire with a diameter of 100 μm, and two NdFeB permanent magnets with a dimension of 4 mm × 4 mm × 3 mm.

The self-folding unit experiences four stages during the self-folding process. As shown in Figure 5c, in stage 1: the distance between the left magnet and the right magnet was relatively far (the initial distance was 16.39 mm), and the attractive force between the opposite poles was less than the friction; stage 2: after the electrical current was applied, the nickel-chromium alloy wire rises in temperature, and the heat-sensitive sheet retracted and pulled the left and the right magnets close to each other; stage 3: as the distance of magnets decreased, the attraction between the two magnetic poles increased rapidly. When the distance was less than the threshold, the attractive force was greater than the friction, and the two magnets attracted rapidly; stage 4: the micro magnets were in contact with each other, and the self-folding unit finished its mission. Finally, the sensor was self-assembled.

Figure 5d,e shows the experimental result of the self-folding process. For a single self-folding unit, when a 1.5 Amp current was continuously applied to the nickel-chromium alloy wire with a DC voltage stabilizing source (MP1005D, MAISHENG, Dongguan, Guangdong, China), the heat-sensitive sheet began to retract after 2 s, and the two micro magnets were in contact with each other after 5.5 s. We used three self-folding units to assemble the miniature soft sensor (a total of 12 creases). We connected the nickel-chromium alloy wires of the three in series and applied a 1.5 Amp current to heat up the heat-sensitive sheet. After 5 s of heating, the heat-sensitive sheet started to retract, taking 17 s to complete the self-assembly process.

The self-folding technique completed the self-folding process of 12 creases in 17 s, with an average time of 1.42 s for each crease. In addition, the self-folding process was activated by electrical current and did not rely on complicated equipment such as lasers, ovens, or other special equipment.

### 2.5. Experimental Setup

**Figure 6:**
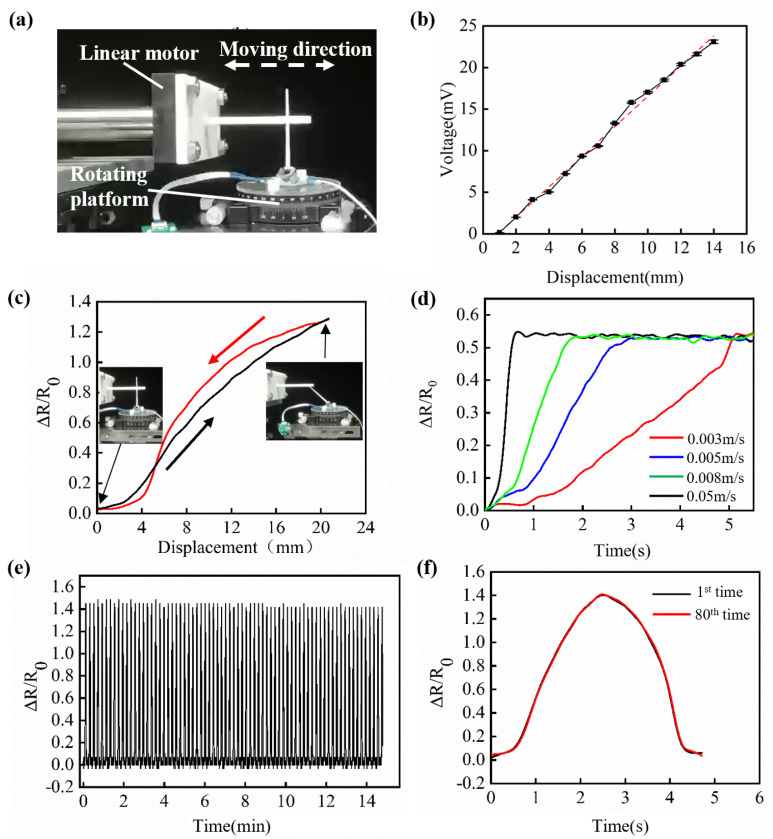
Sensor performance of the miniature soft sensor. (**a**) The experimental setup. (**b**) Linearity results from the sensor. (**c**) Sensor loading and unloading performance. The black line represents the sensor resistance for displacement from 0 to 22 mm, and the red line represents the sensor resistance for displacement from 22 to 0 mm. (**d**) Sensor dynamic response. Different color curves indicate the change of the sensor’s resistance value under different actuation speeds of the linear motor (**e**) A repeating test shows that the miniature soft sensor maintains high reliability after pushing and releasing 80 times. (**f**) Comparison of the first and the 80th resistance change curves.

The experimental setup is shown in Figure 6a. The miniature soft sensor was fixed on the rotating platform. The linear motor (Lin-Mot, Spreitenbach, Switzerland) was used to load the sensor beam tip, and the change of loading directions was obtained by varying the angle of the rotating platform. The speed and displacement of the linear motor can be programmed.

A differential amplifying circuit was used to amplify the resistance signal, which enabled amplifying the signal with a tuned amplifying parameter. The sensor data can be measured by a data acquisition circuit consisting of a signal amplifying circuit and an Arduino board, which transmitted the sensor data to a computer via serial communication. The Butterworth filter was used to filter the noise, and then $△R/R_{0}$ was calculated to characterize the relative resistance change. The displacement data of the linear motor and the sensor data were recorded.

To characterize the performance of the sensor, several experiments were implemented, including the sensor’s linearity, output response, signal repeatability, and underwater performance. Each experiment was repeated five times (N = 5).

In the linearity and sensitivity experiments, the rotating platform was kept at the zero position without rotation, and the linear motor was set to move forward for 1 mm at the speed of 10 mm/s and stood still for 2 s as a motion cycle. The experiment consisted of 13 motion cycles with a total displacement of 13 mm. The displacement data and sensor signal were collected at the static stage of each cycle.

In the continuous loading and unloading experiment, the linear motor was set to load a displacement of 21 mm to the sensor’s beam tip at a speed of 5 mm/s. After reaching the set displacement, the linear motor changed direction immediately, pulling the beam back to its initial position at the same speed. We recorded the displacement data of the linear motor and the sensor signal. When the sensor’s response speed was evaluated, the linear motor was programmed to push the sensor’s beam tip at the horizontal speed of 3 mm/s, 5 mm/s, 8 mm/s and 50 mm/s, respectively. The loading displacement was set to 25 mm.

In the signal repeatability experiment, 80 times of reciprocating loading was implemented on the sensor’s beam at the speed of 5 mm/s. The one-way displacement was 20 mm. In the multi-directional sensing experiment, the linear motor was programmed to load a displacement of 20 mm at the sensor’s beam tip at a speed of 5 mm/s. We changed the angle of the rotating platform to 0°, 60°, 120°, 180°, 240°, and 300°. During the experiment, the sensor signal and the corresponding rotating platform angle were recorded, respectively.

In addition, we tested the sensor’s ability to sense the water flow field at different speeds underwater. We created different flow rates by changing the distance between the water outlet and the sensor. The water outlet us inner diameter is 15 mm with a maximum water output of 0.15 L/s. We opened the valve at a constant speed to slowly increase the flow velocity, which took 4 s to reach the maximum speeds of 50 mm/s, 30 mm/s and 20 mm/s (measured by a hall water flow sensor).

## 3. Results

Figure 6b shows the linearity and sensitivity results. When the linear motor pushed the sensor beam tip to move, the folding angle of the soft sensor gradually increased, which caused the cross-sectional area of the liquid metal channel to decrease, and the sensor output voltage increased accordingly. The following equation describes the sensor output voltage and the loading displacement relationship:

(9) $$U=1.82\times s-1.61\;\;\;\;(R^{2}=0.99)$$

where *U* is the output voltage of the sensor in millivolt, and *s* is the loading displacement in millimeter. Further data analysis showed that the linearity of the soft sensor is 0.046; the sensitivity is 1.82 mV/mm, and the resolution can reach 1 mm.

The continuous loading and unloading results are shown in Figure 6c. Here we used $△R/R_{0}$ signal instead of a voltage signal to show the sensor’s mechanical property since this sensor’s essence principle is soft material deforming. The voltage and resistance’s relationship are linear, and the two signals can be converted. At the initial stage, when the linear motor pushed the sensor’s tip, the sensor resistance increased slowly, and at the loading displacement of 21 mm, $△R/R_{0}$ reached a maximum of 1.3. Then the linear motor unloaded at a constant speed, and the sensor resistance gradually decreased. There was a certain degree of inconsistency between the sensor signal during loading and unloading. The resistance change during unloading was more significant than the change during loading before the displacement decreasing to 5 mm. However, the sensor resistance decreased rapidly when the displacement reached 5 mm during unloading.

The dynamic performance of the sensor is shown in Figure 6d. Under different loading speeds, the sensor had different output signals, which had a different slope of the output signal corresponding to the loading speed. As shown in the figure, when the loading speed was 50 mm/s, the sensing signal maintained a good response. In addition, the maximum displacement of the linear motor was 15 mm despite the different pushing speeds. When a fixed displacement was applied to the sensor beam tip, results show that loading speed had little influence on the sensor signal.

Repeatability is another critical indicator to measure sensor performance. Figure 6e shows the results of the sensor repeatability. In the process of 80 cycles of loading and unloading, the increase of the sensor resistance value did not decline, and the peak value of $△R/R_{0}$ was around 1.4. In addition, comparing the results of the first loading-unloading experiment with the 80th experiment, Figure 6f shows that the resistance curves of the two times almost overlap, indicating that the sensor’s output characteristics did not change after the 80th repeated experiment. We took the loading times when the sensor signal declined to 95% of the initial signal as the sensor lifetime. In this experiment, as loading 200 times, the sensor signal declined 5% compared to the initial signal, and the measuring error reached 1 mm. The life span of the sensor will increase as the loading displacement is less than 20 mm, considering the mechanical property of the soft material.

**Figure 7:**
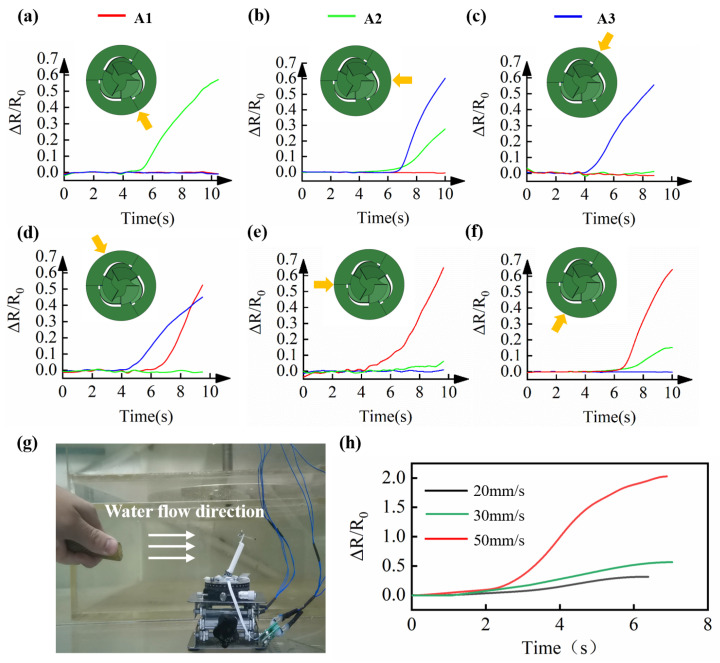
Loading direction and velocity sensing capability of the miniature soft sensor. (**a**–**f**) The miniature soft sensor perception of external load in different directions. Yellow arrows indicate the direction of the load. (**g**) Experimental setup of the water flow perception experiment. (**h**) Sensor signal under different water flow speeds.

Figure 7a–f shows that the sensor can measure stimuli from different directions. The illustration in Figure 7a–f shows the top view of the sensor, and A1, A2, and A3 represent the three sensing units at the corresponding positions, respectively. According to the three signal values of A1, A2, and A3, with the sensor characterization result and the kinematic model, six external load directions can be distinguished. For example, when green (A2) increased and red and blue (A1 and A3) remained unchanged, it can be analyzed as a stimulus from the five o’clock direction. When green and blue (A2 and A3) increased simultaneously and red (A1) remained unchanged, they can be analyzed as a stimulus from the right. Similar results were found when the stimuli were applied from the other four directions.

Furthermore, we tested the sensing performance underwater. As shown in Figure 7g,h, the sensor can distinguish three external flow velocities of 20 mm/s, 30 mm/s and 50 mm/s, and the slope of the signal reflected water flow velocity increasing from 0 mm/s to a steady-state velocity (20 mm/s, 30 mm/s and 50 mm/s). The flow had an impact on the upstream surface of the sensor. The faster the flow velocity, the greater the impact. The force of the flow was balanced with the restoring force generated by the deformation of the soft material and finally reached equilibrium. We tested the minimum flow rate of 20 mm/s, with the change of sensor resistance $△R/R_{0}$ of 2.0, indicating a minimal flow rate that the sensor can accurately perceive.

## 4. Discussion

In this paper, we designed a millimeter-scale, three-dimensional-shaped soft sensor inspired by origami structure. The sensor was fabricated with the planar manufacturing process and can self-assemble by applying electrical currents. According to the experimental results, the sensor’s sensitivity is 1.82 mV/mm, linearity 0.046, displacement range ±20 mm, and resolution 1 mm. Meanwhile, the sensor shows good repeatability. In addition, the underwater experiment shows that the sensor can measure a minimum velocity of 20 mm/s, which provides a new approach for underwater sensing. Our work shows the possibility of applying millimeter-scale soft sensors for future underwater robots.

Millimeter-scale soft sensing often has limited deformation capability and measurement limitations [22,23,24]. In our work, the miniature soft sensor can withstand large deformation through soft material and origami mechanism design. Meanwhile, according to the kinematic model, the resulting displacement can be calculated.

Fabrication of millimeter-scale sensors is another challenge. We introduced a sensing unit on origami structure, which provides sensing ability with excellent deformation capability. As for fabrication, all the steps are based on planar manufacturing, and the sensor could self-assemble by applying current to self-folding units. In order to increase productivity, mass production can be applied. Through the fabrication process above, the minimum crease width is 3 mm. Compared with the previous studies [29,31,32,33], our self-folding technique only takes 17 s to fold 12 folding creases, with an average time of 1.42 s for each crease. Total energy consumption is 26.775 J, with an average energy consumption of 2.231 J for each crease.

There are still a few limitations to the current miniature soft sensor. Due to the mechanical property of silicone elastomers, liquid metal micro-channel shows mechanical hysteresis when it withstands the external load. At present, the main way to improve the hysteresis of soft sensors is to reduce the influence of elastomer viscoelasticity by changing the structure [41,42,43]. In future work, we will explore the low hysteresis performance of flexible sensing from the shape of the liquid metal micro-channel, the composition of the liquid metal, and the sensor structure. Furthermore, the range of the sensor is determined by the mechanical design. Our design’s folding angle can reach 70 degrees without compromising sensor linearity. Those limitations may compromise sensor accuracy when measuring the directional angle of the displacement. The error of the angle is ±15 degrees. In the future, establishing a more comprehensive physics model would further complement the sensor’s model.

## Figures and Tables

**Table 1:** Parameters of the linkage.

Symbol	Value	Description
*L*	6 mm	Length of ${\mathrm{OI}}_{i}$
*R*	6 mm	Length of ${\mathrm{OO}}^{{}^{\prime }}$
$\beta_{1}$	0°	The angle between the projection of ${\mathrm{OI}}_{1}$ on the Oxy plane and the negative direction of the *x*-axis
$\beta_{2}$	120°	The angle between the projection of ${\mathrm{OI}}_{2}$ on the Oxy plane and the negative direction of the *x*-axis
$\beta_{3}$	240°	The angle between the projection of ${\mathrm{OI}}_{3}$ on the Oxy plane and the negative direction of the *x*-axis

## Supplementary Materials

The following supporting information can be downloaded at: https://www.mdpi.com/article/10.3390/mi13081188/s1, Video S1: Self-folding process.
